# Enhancing antisense efficacy with multimers and multi-targeting oligonucleotides (MTOs) using cleavable linkers

**DOI:** 10.1093/nar/gkv992

**Published:** 2015-10-07

**Authors:** Romesh R. Subramanian, Mark A. Wysk, Kathleen M. Ogilvie, Abhijit Bhat, Bing Kuang, Thomas D. Rockel, Markus Weber, Eugen Uhlmann, Arthur M. Krieg

**Affiliations:** 1 RaNA Therapeutics LLC, 790 Memorial Dr., Suite 203, Cambridge, MA 02139, USA; 2 Pfizer Worldwide Research and Development, 9381 Judicial Dr., Suite 200, San Diego, CA 92121, USA; 3 Coley Pharmaceutical GmbH, Merowingerplatz 1a, 40225 Dusseldorf, Germany

## Abstract

The *in vivo* potency of antisense oligonucleotides (ASO) has been significantly increased by reducing their length to 8–15 nucleotides and by the incorporation of high affinity RNA binders such as 2′, 4′-bridged nucleic acids (also known as locked nucleic acid or LNA, and 2′,4′-constrained ethyl [cET]). We now report the development of a novel ASO design in which such short ASO monomers to one or more targets are co-synthesized as homo- or heterodimers or multimers via phosphodiester linkers that are stable in plasma, but cleaved inside cells, releasing the active ASO monomers. Compared to current ASOs, these multimers and multi-targeting oligonucleotides (MTOs) provide increased plasma protein binding and biodistribution to liver, and increased *in vivo* efficacy against single or multiple targets with a single construct. *In vivo*, MTOs synthesized in both RNase H-activating and steric-blocking oligonucleotide designs provide ≈4–5-fold increased potency and ≈2-fold increased efficacy, suggesting broad therapeutic applications.

## INTRODUCTION

Antisense oligonucleotide (ASO) therapeutics are emerging as the third platform for drug development after small molecules and biologics, with more than 100 compounds in clinical development. There are two standard single-stranded ASO designs that act by different mechanisms of action (1–3). First, ‘gapmer’ ASOs contain a DNA center that activates RNase H to cleave the target RNA: this core generally is flanked by 5′ and 3′ wings of nucleotides with ribose modifications such as 2′- methoxyethyl (MOE) or LNA which do not support RNase H cleavage, but which confer an increased affinity for hybridization. Second, ‘shortmer’ and ‘mixmer’ ASOs act by steric blocking, and contain ribose modifications distributed in such a manner that the ASO does not activate RNase H. Historically, the standard ASO gapmers were ≈20 nucleotides in length which was derived based on *in vitro* transfection experiments in which ASOs of at least 20 bases in length were found to be more effective than shorter ASOs. More recently, several groups have reported that the incorporation of LNA or cET makes it possible to use shorter gapmer ASOs of less than 15 nucleotides in length (4–7): steric blocking shortmer ASOs can be as short as 8 nucleotides long (8).

One of the major factors affecting ASO pharmacokinetics and *in vivo* efficacy is their plasma protein binding. ‘Standard’ 20mer phosphorothioate (PS) backbone ASO typically are ≈95% protein bound in plasma (i.e. ≈5% unbound) (9). High plasma protein binding of ASO reduces renal excretion of the unbound ASO, the major route of *in vivo* elimination. This high plasma protein binding of PS ASO is low affinity, and therefore facilitates uptake into peripheral tissues via higher affinity receptors (10). While investigating the plasma protein binding and other pharmacological properties of short LNA ASO, we observed that a 13mer ASO PS LNA gapmer was more than 20% free (unpublished data), which is consistent with previous results on the length dependence of ASO protein binding (9,11). We hypothesized that such low plasma protein binding would cause faster renal excretion *in vivo*, decreasing therapeutic efficacy. We further hypothesized that the plasma protein binding and the *in vivo* activity of short ASOs could be increased by synthesizing them as homodimers or trimers connected via a linker. Finally, we extended this new ASO design to heterodimers in which ASO to two different targets are linked together, creating a new class of ASO construct that we have named multimers and multi-targeting oligonucleotides (MTOs), which we now demonstrate to have improved *in vivo* pharmacologic activity as compared to standard short ASO designs.

## MATERIALS AND METHODS

### Hepatocyte lysate assay for ASO stability

Bulk liver homogenate was prepared by disruption of 200 mg of mouse whole liver in 800 ml of 100 mM Tris-acetate (pH 8.7), 10 mM EDTA. Calibration samples for ASO standard curves for bioanalysis were prepared using 30 ml homogenate (or 5 mg liver) for each sample, spiked with ASO individually in the range of 2000 ng/g to 20 000 ng/g final concentration. Each sample was then extracted with 100 ml phenol, the aqueous phase was precipitated (2x) with 200 ml cold isopropanol and the resulting pellet was resuspended in 200 ml of 100 mM Tris acetate buffer (pH 8.7). Thirty (30 ml) of each sample was then analyzed for ASO concentration using a hybridization assay. Excess complementary 5′ FAM labeled probe ASO (5 ng total) was added, gently vortexed and then incubated at 95°C for 5 min, then 55°C for 10 min, prior to loading for capillary electrophoresis with laser-induced fluorescence (Spectrumedix Reveal, Transgenomic). To determine ASO stability in the test matrix, liver homogenate samples were incubated with ASO at concentrations in the high end of the linear range for time periods up to 28 days in a similar manner, except for an overlay of mineral oil and parafilm of the tube to prevent evaporation during extended incubation at 37°C. At each indicated timepoint, the assigned tube was removed and maintained at −20°C prior to extraction, hybridization and ASO quantification using capillary electrophoresis. After capillary electrophoresis, the fluorescent species within each capillary were observed and recorded for fluorescent intensity (three capillaries were assessed and averaged for each sample result). Calibration curves (hyperbolic) were created by plotting the fluorescent intensity of Hybrid peak heights in each capillary set for each calibration standard. Sample results were treated similarly, and the fluorescent intensity was then back-calculated to the established curve to obtain the reported result.

### *In vitro* experiments with ASO to ApoB and/or ApoC3

Human hepatocarcinoma cells (Hep3B) were seeded at 3–10 E3 cells/well (1–3 days prior to treatment) into 96 well multi-titer plates yielding 70–80% confluence on the day of treatment. For assays using lipotransfection delivery techniques, cells were incubated with indicated concentrations of ASO formulated with 0.3 μl Lipofectamine 2000 (L2k) for 48 h in Earle's Balanced Salt Solution (Lonza, Verviers, Belgium) with L-glutamine (2 mM). For free uptake (‘gymnotic delivery’) (12), the cells were not transfected with the ASO, but instead were incubated with indicated concentrations of unformulated ASO in MEM with high glucose (6 g/l; Invitrogen, Carlsbad, CA, USA) without L-glutamine for 8 days. Following the treatment period mRNA levels of target and reference (a housekeeping gene) mRNA was determined by the Quanti Gene 2.0 Assay (Affymetrix, Santa Clara, CA, USA) according to the manufactures standard protocol. Human ApoB/ApoC3 probes and housekeeping gene PPIB probes were purchased from Affymetrix. Prior to lysis, cell viability was analyzed by Cell Titer Blue Assay (Promega, Madison, WI, USA). The data were normalized against housekeeping gene PPIB.

### *In vivo* experiments

mIR 122 targeting LNA oligonucleotides (either monomers or multimers), APOC3/ApoB ASO heterodimers or non-targeting ASO (negative control) were formulated in sterile PBS (vehicle, pH7.4) immediately before subcutaneous (sc) injection in the flank in a volume of 100ul. *In vivo* activity of miR122 targeting LNA oligonucleotides was assessed in female C57bl/6J mice (10–12 weeks old), whereas heterodimers of a human APOC3 ASO linked to an ApoB ASO were assessed in male and female human APOC3 transgenic mice [B6;CBA-Tg(APOC3)3707Bres/J (stock 006907), Jackson Laboratories] which were 9–18 weeks old at termination. All procedures involving live animals were reviewed and approved by the appropriate Institutional Animal Care and Use Committee prior to experimentation.

For experiments using miR-122 targeting oligonucleotides, mice were dosed with 1, 3 or 10 mg/kg of monomer oligo (adjusted for linker length in order to maintain molar equivalence), on days 1 and 4 and euthanized on day 7 by CO_2_ inhalation, at which time liver and kidneys were collected, flash frozen in liquid nitrogen and stored at −80°C for subsequent analyses. Total RNA including miRNAs were prepared from liver samples using a miRNeasy kit (Qiagen). Subsequently cDNA was generated and expression of genes (mRNA) normally inhibited by miR-122 were assessed by qRT-PCR to ascertain efficacy of decreasing miR-122 binding.

In a first experiment evaluating the effects of the ApoC3/ApoB gapmers, groups of 2 male and 2 female transgenic mice were terminated on days 1, 3, 7, 14 and 29 after treatment with a single intravenous administration of the ApoB/ApoC3 heterodimer with the cleavable linker (MTO; Table 1) at 0.3, 1, 3 or 10 mg/kg or negative control ASO (10 mg/kg) or saline (0 mg/kg) in a volume of 5 ml/kg. In a second experiment, groups of 6–7 mice were treated once intravenously with equi-molar amounts of the indicated ASO, euthanized 3 or 14 days later by CO_2_ inhalation, and blood was obtained by cardiac puncture (0.5–1 ml). Depending on the experiment, liver or liver and kidney were dissected, weighed and a fragment saved in a labeled histology cassette snap frozen by immersion in liquid nitrogen. Each blood sample was divided in half. Serum was prepared in serum separator tubes which were allowed to clot for 4 h on ice. Plasma was prepared in EDTA-containing tubes which were maintained on ice until processed. Tubes were spun at 10 000 rpm for 5 min at 4°C and supernatants collected. Tissue samples were maintained at −80°C for subsequent analyses.

**Table 1:** Sequences of antisense oligonucleotides (ASOs) and controls

Name/ sequence #	Purpose	Sequence
Monomer	miR-122 shortmer monomer	ßC*ßA*ßC*ßA*ßC*ßT*ßC*ßC
Dimer	miR-122 shortmer MTO dimer	ßC*ßA*ßC*ßA*ßC*ßT*ßC*ßC–T-T-T-T-ßC*ßA*ßC*ßA*ßC*ßT*ßC*ßC
Trimer	miR-122 shortmer MTO trimer	ßC*ßA*ßC*ßA*ßC*ßT*ßC*ßC–T-T-T-T-ßC*ßA*ßC*ßA*ßC*ßT*ßC*ßC–T-T-T-T-ßC*ßA*ßC*ßA*ßC*ßT*ßC*ßC
Monomer control	miR-122 shortmer monomer control	βC*βG*βC*βA*βC*βC*βC*βC
Dimer control	miR-122 shortmer dimer control	βC*βG*βC*βA*βC*βC*βC*βC-T-T-T-T-βC*βG*βC*βA*βC*βC*βC*βC
Trimer control	miR-122 shortmer trimer control	βC*βG*βC*βA*βC*βC*βC*βC-T-T-T-T-βC*βG*βC*βA*βC*βC*βC*βC-T-T-T-T-βC*βG*βC*βA*βC*βC*βC*βC
128	miR-122 gapmer monomer	ßC*ßA*ßT*T*G*T*C*A*C*A*C*T*ßC*ßC*ßA
129	miR-122 gapmer MTO dimer	ßC*ßA*ßT*T*G*T*C*A*C*A*C*T*ßC*ßC*ßA-T-T-T-TßC*ßA*ßT*T*G*T*C*A*C*A*C*T*ßC*ßC*ßA
130	Control for miR-122 gapmer monomer	ßT*ßG*ßA*A*G*G*T*T*C*C*T*C*ßC*ßT*ßT
131	Control for miR-122 gapmer dimer	ßT*ßG*ßA*A*G*G*T*T*C*C*T*C*ßC*ßT*ßT-T-T-T-TdßT*ßG*ßA*A*G*G*T*T*C*C*T*C*ßC*ßT*ßT
CTRL	Negative Control ASO gapmer	ßT*ßA*ßA*T*Z*G*T*Z*G*A*T*ßA*ßZ*ßZ
ApoB / 13	ApoB ASO monomer gapmer	ßG*ßZ*A*T*T*G*G*T*A*T*ßT*ßZ*ßA
ApoC3 / 30	ApoC3 ASO monomer gapmer	ßG*ßZ*dA*dC*dT*dG*dA*dG*dA*dA*dT*dA*ßZ*ßT
MTO / 21	ApoC3 / ApoB MTO heterodimer	ßG*ßZ*A*C*T*G*A*G*A*A*T*A*ßZ*ßT-T-T-T-T-ßG*ßZ*A*T*T*G*G*T*A*T*ßT*ßZ*ßA
MTPS / 59	ApoC3 / ApoB MTPS heterodimer	ßG*ßZ*A*C*T*G*A*G*A*A*T*A*ßZ*ßT*T*T*T*T*ßG*ßZ*A*T*T*G*G*T*A*T*ßT*ßZ*ßA
18	ApoB homodimer MTO	ßG*ßZ*A*T*T*G*G*T*A*T*ßT*ßZ*ßA-T-T-T-T-ßG*ßZ*A*T*T*G*G*T*A*T*ßT*ßZ*ßA
31	ApoB mismatched negative control monomer	ßZ*ßG*T*C*T*A*T*G*T*A*ßT*ßA*ßG

Z = 5-methyl cytidine, ß = locked nucleic acid (LNA), * indicates phosphorothioate internucleotide linkage, - indicates phosphodiester internucleotide linkage, all bases are deoxy unless otherwise specified.

### Quantification of target mRNAs by qRT-PCR

For mir122 experiments, total liver RNA was prepared using the miRNEasy kit (Qiagen) according to manufacturer's recommendations and used for cDNA synthesis with the High Capacity cDNA Reverse Transcription kit (Life Tech). qRTPCR was performed on a StepOnePlus Real Time PCR System with primer probe sets purchased from Life Tech (mAldoA, Mm00833172_g1; mBCKDK, Mm00437777_m1). Expression values were calculated using the comparative threshold cycle method normalized to mouse GusB (Mm01197698_m1).

For ApoC3/ApoB experiments, total liver RNA was isolated in TRIzol reagent (Ambion) from snap frozen tissue homogenized in Fastprep24 Lysing Matirx D tubes (MP Biomedicals). Trizol-chloroform extraction was followed by further purification using a column-based method (Qiagen, RNeasy) as per manufacturer's instruction. Purification included treatment with Dnase I for 15 min at room temperature (Qiagen, Rnase-Free Dnase). RNA quantity and purity were evaluated spectophotometrically by readings at 260 nm and 280 nm (Nanodrop). Liver fragments were lysed with RLT buffer and QIAshredder columns (Qiagen), and then purified by Rneasy columns as indicated above. Samples were amplified as per manufacturer's instructions (Qiagen, Quantitect Probe RT-PCR kit). Quantitative real-time PCR (qRT-PCR) was performed in a 7900HT Fast Real-Time PCR System (Applied Biosystems). All samples were analyzed in triplicate in Microamp Optical 384well reaction plates (Applied Biosystems) and normalized with Gapdh signal as the internal control. Results are expressed as fold induction relative to vehicle-treated samples.

Data for each endpoint were analyzed by one or two-way ANOVA using ‘time’ and/or ‘treatment’ as the variables in GraphPad Prism software. Bonferroni post-hoc tests were conducted when significant main effects (*P* ≤ 0.05) were observed.

### Serum lipid measurements

Serum biomarkers were measured on an Advia 1200 clinical chemistry analyzer using reagents purchased from Siemens Diagnostics.

### Determination of tissue concentrations of ApoB/ApoC3 heterodimers and monomer

Liver and kidney homogenate was prepared from mice treated with heterodimeric or monomeric ASOs. Tissue samples collected at specified time points were minced and weighed in ready-to-use Lysing Matrix D tubes containing 1.4 mm ceramic spheres beads (Catalogue# 6913–100, MP Biomedicals). Dnase/RNAse free water (Catalogue # SH30538.02, Thermo) was added to the tube with ratio of 5 or 10 ml per g of tissue. Each tissue sample was mixed and homogenized using a MP Biomedicals Fast Prep-24 at 4°C for 20 s twice. The tissue homogenate was stored in freezer or kept on ice before analyzed with the hybridization assay. Standards and assay quality controls (QCs) were prepared in K2 EDTA plasma or control tissue matrix and diluted serially in 2-fold steps from 100 ng/ml to 0.098 ng/ml. The QCs were set at 50 ng/ml, 40 ng/ml, 10 ng/ml, 1 ng/ml and 0.4 ng/ml. The standards and QCs were analyzed by the hybridization assay with the samples.

Hybridization assays were developed to measure the concentrations of the heterodimers with the cleavable and the stable phosphorothioate linkers as well as the ApoB monomer ASO in plasma and homogenates of liver and kidney following the basic approach described previously for other oligonucleotides (13). Briefly, complementary hybridization probes to the heterodimeric ASOs were designed and custom synthesized with LNA-modified phosphodiester backbones (BioSpring GmbH) using methods above. The capture probes contained an amino linker (C12-amino) and Spacer-18s (hexaethyleneglycol phosphate, PEG-282) at the 5′-end. The detection probes contain Spacer-18s at the 3′-end of the specific probe sequence and were biotin labeled at the 3′-end (specific sequences of capture and detection probes are shown below).

The hybridization assays were performed in DNA-Bind plates (96-well) (Catalogue #2505, Costar) coated overnight at 4°C with 100 μl of 50 nM capture probes in HEPES/1mM Na_2_ EDTA buffer. The plates were then washed three times with wash buffer (Tris Buffer/0.1% Tween 20) and incubated in blocking buffer (PBS/3% BSA) for 1–2 h. 30 μl of Samples, Standards and QCs were mixed with 270 μl of 50 nM detection probe in hybridization buffer (4X SSC/0.5% Sarkosyl) in Costar cluster tubes and two 100 μl aliquots from the mixture were transferred into 96-well PCR plate and denatured on the thermocycler for 12.5 min at 95°C. After the samples were cooled to 40°C, they were transferred to DNA-Bind plate already coated with capture probe. The plate was sealed and incubated at 40°C for 2 h. Following the hybridization, Poly-HRP Streptavidin conjugate (Catalogue # N200, Thermo) at 1:10 000 dilution in Poly –HRP dilution buffer (Catalogue # N500, Thermo) was added. Color development was initiated by adding SureBlue TMB substrate (Catalogue #52–00–00, KPL) and stopped with stop reagent for TMB substrate (Catalogue # S5814, Sigma).

### Synthesis of oligonucleotides

Oligonucleotides targeting miR-122 were purchased from BioSpring GmbH (Frankfurt, Germany). The other homo- and heterodimers were synthesized using a standard synthesis protocol on an AKTA oligopilot 10 Plus synthesizer. The oligonucleotides were cleaved from solid support using a solution of ammonium hydroxide and ethanol (3:1) at 55°C for 17 h. The crude oligonucleotides were purified in a two-step IEX-purification procedure using a Source 30Q column and buffer system containing sodium hydroxide. The mass spectrometer analysis was done using ESI-MS and the purity was established using HPLC. The endotoxin levels were measured by LAL.

Capture and detection probes were designed for heterodimer capture [5′-(C12-amino)(Spacer-18)(Spacer-18)-ßG-ßC-ßA-ßA-ßA-ßA-ßA-ßG-3′]; heterodimer detection [5′-ßT-ßC-ßA-ßG-ßT-ßG-ßC-(Spacer-18)(Spacer-18)(dT-biotin)(biotin TEG)-3′]; ApoB monomer capture [5′-(C12-amino)(Spacer-18)(Spacer-18)-ßT-ßG-ßA-ßA-ßT-ßA-ßC-3′] and ApoB monomer detection [5′- ßC-ßA-ßA-ßT-ßG-ßC-(Spacer-18)(Spacer-18)(dT-biotin)(biotin TEG)-3′]) and were synthesized using a standard protocol on an AKTA oligopilot 10 Plus synthesizer. Oligonucleotides were cleaved from solid support using a solution of ammonium hydroxide and ethanol (3:1) at 55°C for 17 h and purified in a two-step RP-/IEX-purification procedure. The RP-purification was by applying a TEAA-containing buffer system and the IEX purification was carried out at physiological conditions. The mass spectrometer analysis was done using ESI-MS and the purity was established using HPLC.

## RESULTS

### Design of multimers and multi-targeting oligonucleotides (MTOs)

**Figure 1.**
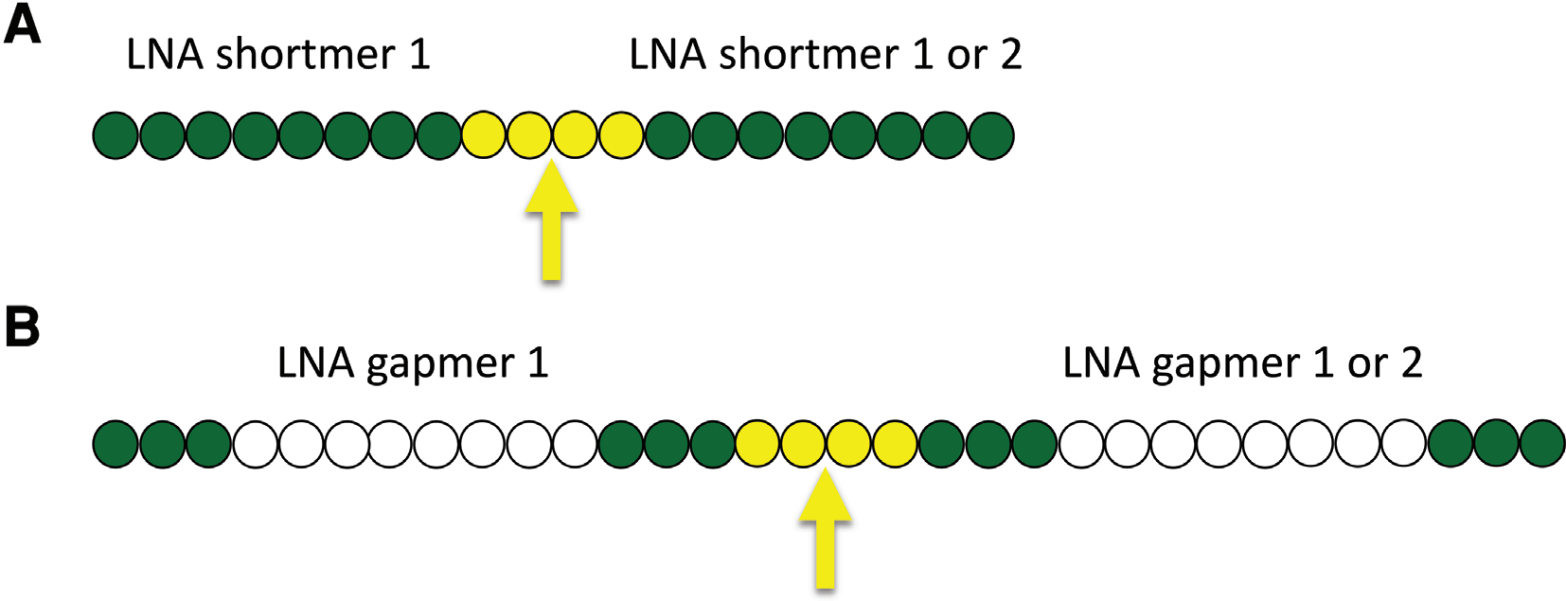
General schematics for MTO gapmer design. An MTO can be designed to include nearly any two shortmers (**A**), mixmers (not depicted), or gapmers (**B**) and may be linked via any linker that is cleaved in endosomal compartments (yellow arrow indicates region of cleavage). A simple linker is 4 nucleosides linked by phosphodiester bonds. Green circles denote nucleoside modifications that do not activate RNase H (e.g. LNA) while open circles do support RNase H–mediated cleavage of a target RNA. For gapmers, the figure is drawn to indicate 14mer components, but other lengths also can be used.

Because short phosphorothioate backbone ASO have lower plasma protein binding than longer ASO (9,11) we hypothesized that they would undergo greater renal clearance *in vivo*, with a corresponding reduction in uptake into liver and other tissues, and a loss of therapeutic efficacy. We further hypothesized that short ASO uptake into the liver and their *in vivo* activity would be increased by synthesizing short ASO in the form of dimers or trimers, linked via phosphodiester linkages to allow the ASO to be cleaved into the monomers after cellular uptake (Figure 1).

### MTO shortmers and gapmers against mir122

**Figure 2.**
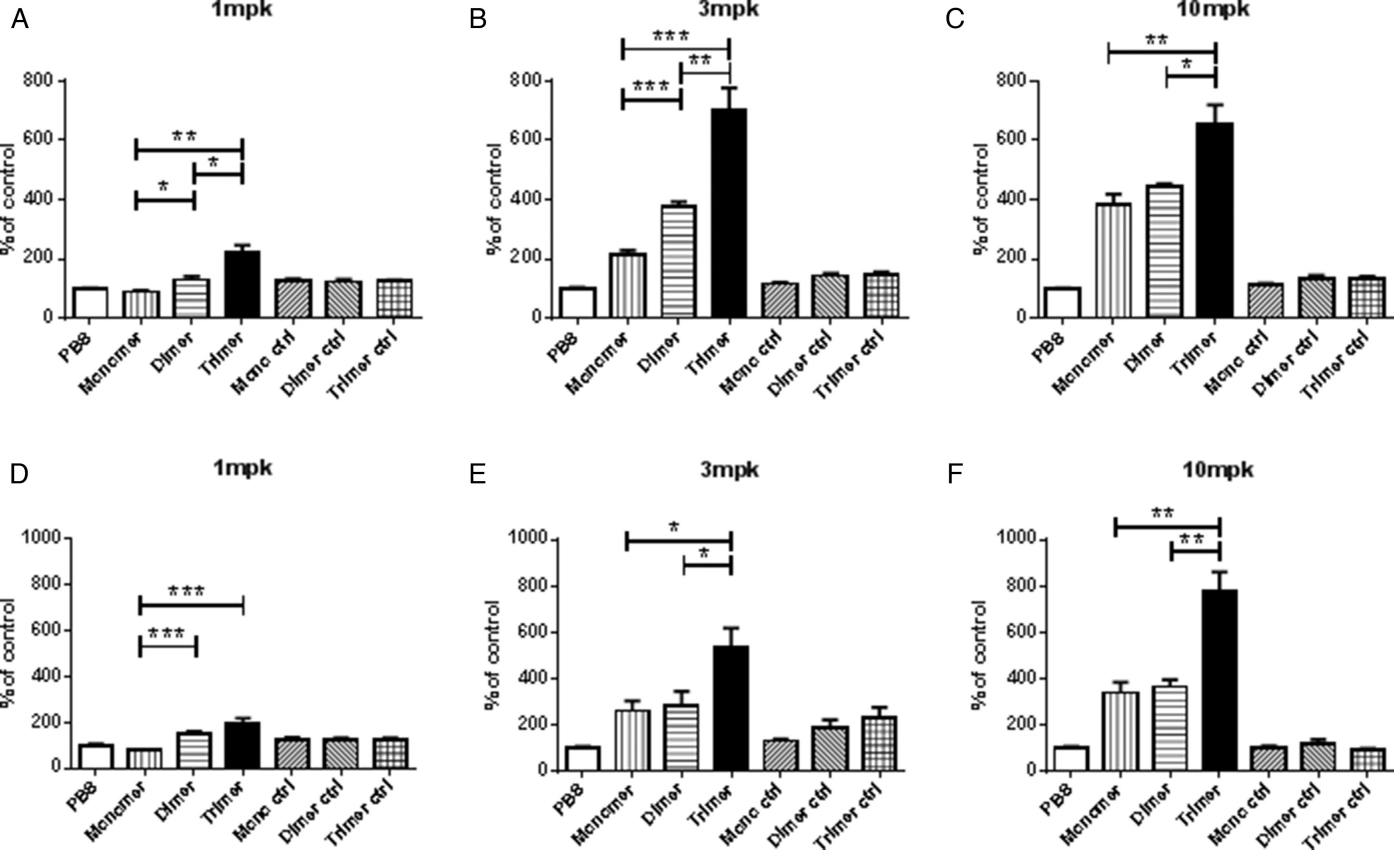
*In vivo* efficacy of shortmer MTOs targeting miR-122. (**A**–**F**) Female C57BL6/J mice were dosed with 1, 3, or 10 mg/kg (of monomer equivalent: doses were adjusted for linker length to deliver molar equivalence of monomers) of fully LNA-modified phosphorothioate oligonucleotides with sequences as in Table 1 with anti-miR-122 shortmers (either monomers or MTO dimers or trimers) or non-targeting monomer, dimer or trimer controls containing the same pattern of nucleoside modifications ASO (negative control) sc, on days 1, 2 and 3 and liver harvested on day 7 for RNA extraction and quantification of the miR-122 target genes BCKDK (A, B, C) and AldoA1 (D, E, F) to ascertain efficacy of miR-122 antagonism. Three experiments were performed with similar results. *P < 0.05; **P < 0.005; ***P < 0.0005.

In order to test the properties of the MTO design, we performed a series of studies with various ASO, starting with a previously reported 8mer shortmer blocking miR-122 (8,14). This 8mer was synthesized as a monomer, and as an MTO homodimer or trimer linked via nuclease-sensitive phosphodiester DNA (Table 1). The *in vivo* efficacy of these ASO was tested by measuring the de-repression of expression of the miR-122-regulated genes BCKDK and AldoA1 in the livers of normal mice dosed subcutaneously. The 8mer demonstrated a dose-dependent induction of BCKDK expression up to ≈4-fold at the highest dose of 10 mg/kg (Figure 2). A homodimer of this same oligo showed increased *in vivo* efficacy, reaching the same 4-fold induction of expression of the BCKDK target at a dose of just 3 mg/kg, and a homotrimer of the steric blocker had an even greater activity, reaching an ≈7-fold induction of the target at the 3 mg/kg dose. Thus, the MTO design in this case provides a greater than 3-fold improvement in *in vivo* potency compared to a traditional monomeric shortmir, with the added potential benefit of an increased peak induction of BCKDK expression to almost twice that achieved with the monomer. A second target gene of mir122, AldoA1, was also highly upregulated with shortmers targeting miR-122, confirming the increased potency of the MTO designs compared to monomers (Figure 2).

**Figure 3.**
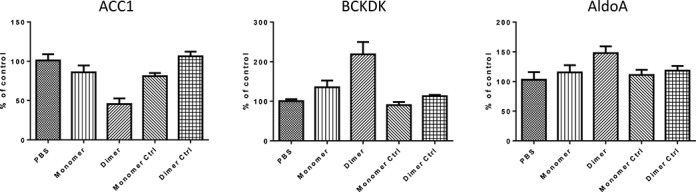
*In vivo* efficacy of gapmer MTOs targeting miR-122. C57BL6/J female mice (5 mice / group) were dosed as indicated with PBS or anti-miR-122 gapmer monomer (oligo #128) or dimer (oligo #129) or with the monomer control (Ctrl) (oligo #130), or dimer control (oligo #131) sc on days 1 and 4 at 10 mg/kg, and the livers harvested on day 7 for RNA extraction and quantification of the miR-122 target genes ACC1, BCKDK and AldoA1 to ascertain efficacy of miR-122 antagonism. Data represent the mean ± SEM. Three experiments with these gapmers were performed with similar results. *P < 0.05.

To determine whether the increased efficacy of the cleavable MTO design could be extended to RNase H-activating gapmer ASO, we designed and synthesized a 15 nucleotide long conventional gapmer against miR-122 (ASO 128 in Table 1), and compared its activity *in vivo* to that of an MTO containing two copies of the gapmer (ASO 129 in Table 1, data in Figure 3) using the same assay for induction of BCKDK and AldoA1 and also showing the suppressive effect on ACC1, which is regulated in the opposite manner by miR 122 inhibition using gapmers (15). By these readouts, the MTO dimer at 10 mg/kg achieved significantly greater miR-122 inhibition than the monomer. These studies demonstrated that the *in vivo* activity of a shortmer or gapmer ASO can be dramatically increased by synthesizing it as an MTO, simply by connecting two or more monomers with four or five phosphodiester-linked nucleosides.

### MTO heterodimers targeting both ApoB and ApoC3

**Figure 4.**
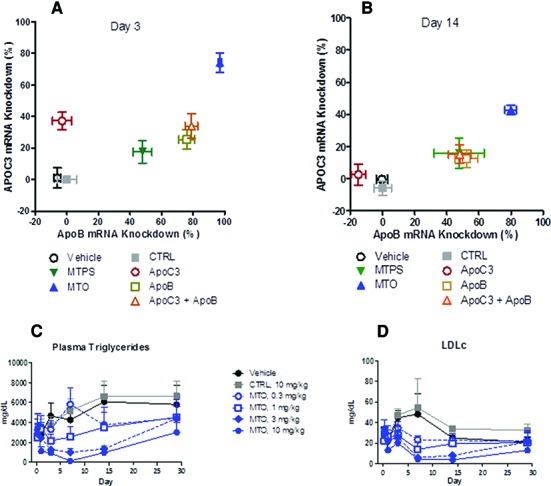
*In vivo* efficacy of monomer and heterodimer ASOs at Day 3 and Day 14. Equimolar doses (0.3 mmol/kg) of the two APOC3/ApoB heterodimers (≈3 mg/kg) with a phosphodiester linker (MTO) or phosphorothioate linker (MTPS), the monomers alone or in combination (≈1.3 mg/kg each to maintain equimolar delivery of monomer compared to the heterodimers with linkers), or vehicle or oligonucleotide controls (≈1.4 mg/kg) were administered by single IV injection to human APOC3 transgenic mice (6–7 mice/group). A non-targeting ASO (CTRL) and sterile saline (Vehicle) served as negative controls. Liver tissue samples were obtained 3 or 14 days post-administration and analyzed by qRT-PCR for ApoB and ApoC3 mRNA levels and data plotted as% knockdown, with mouse ApoB mRNA on the x axis and human APOC3 (i.e. the transgene) on the y axis (**A**, **B**). To determine the dose-dependent changes in serum lipids following MTO treatment, human ApoC3 transgenic mice (2 male and 2 female/group) were injected sc with PBS, negative control MTO at 10 mg/kg (CTRL), or the heterodimer MTO at the indicated doses on day 0 (**C**, **D**). Serum was collected at the indicated time points for measurement of triglyceride and LDLc concentrations (liver target mRNA data for these mice are shown in Figure 5).

A further advantage of the MTO design would be in the development of heterodimers or heteromultimers to effectively inhibit two or more targets with a single ASO molecule. To investigate this type of MTO, we selected a published phosphorothioate backbone 13mer LNA targeting apolipoprotein B (ApoB) (6) and synthesized heterodimer MTOs linking this 13mer to a newly developed 14mer LNA gapmer targeting apolipoprotein C3 (ApoC3) (5,16). To determine whether the cleavable phosphodiester linker was required for the improved *in vivo* activity, we also synthesized a phosphorothioate-linked multi-targeting ASO (‘MTPS’). The *in vivo* efficacy of the various gapmer ASO heterodimer designs administered at equimolar doses (0.3 μMol/kg; ≈3 mg/kg for the heterodimer) via single IV injection was evaluated using human APOC3 transgenic mice. The MTO with an endonuclease cleavable linker was markedly superior to all other treatments on both day 3 and day 14 in the inhibition of both target mRNAs (Figure 4A,B). For example, on day 3 the MTO reduced ApoB mRNA in the liver by ≈98% compared to ≈80% reduction with the monomer, and only 50% reduction with the MTPS (Figure 4A). At day 14 the target inhibition was generally lower than at day 3, and only the APOC3/ApoB MTO still significantly suppressed expression of human APOC3 (Figure 4B). The MTO-mediated target knockdown was consistently superior to monomeric ASOs administered either individually or in combination. The monomer targeting ApoB showed a significant off-target effect with suppression of ApoC3 expression, which may be due to the partial complementarity of this monomer to a conserved sequence in ApoC3. In contrast, the monomer targeting ApoC3 was directed at a unique sequence and showed no evident off-target effects. The *in vivo* efficacy of the PS linked heterodimer was significantly inferior to that of the free monomers, and to the phosphodiester-linked MTO. These marked differences in *in vivo* efficacy completely disappeared when the MTO and MTPS were tested by *in vitro* lipofection in human hepatoma cells: both the MTO and MTPS were equally highly active and comparable to the monomers (data not shown).

### Extended duration of efficacy of MTO heterodimers

**Figure 5.**
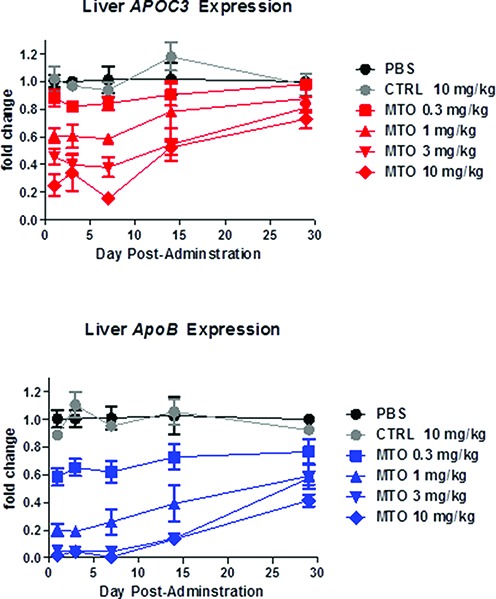
Kinetics of dose-dependent knockdown of ApoB and ApoC3 by heterodimer MTO. Human ApoC3 transgenic mice (2 male and 2 female/group) were injected sc with PBS, negative control MTO at 10 mg/kg (CTRL), or the heterodimer MTO at the indicated doses on day 0. Livers were harvested at the indicated time points for RNA extraction and analysis by qRT-PCR for ApoC3 and ApoB mRNA amounts.

In a separate dose-response and time course experiment the MTO at single doses as low as 1 mg/kg continued to show significant inhibitory effects on both ApoB and ApoC3 liver mRNA until at least 28 days post-administration, the longest time point at which samples were obtained (Figure 5). The biologic consequences of the greater knockdown of APOC3 and ApoB mRNAs by single dose administration of the MTO were demonstrated by dramatic and sustained suppression of circulating serum triglycerides and LDL cholesterol, which were also depressed for more than two weeks (Figure 4C,D).

### Pharmacology and *in vivo* biodistribution of MTO heterodimers

To determine the mechanism for the increased efficacy of the MTO, we assessed the *in vitro* properties and the *in vivo* biodistribution and metabolism of the ApoB/ApoC3 MTO and MTPS heterodimers targeting ApoB and/or ApoC3. As expected, an MTO heterodimer of 31 nucleotides in length had a much higher human plasma protein binding than a 13mer (3% of the 31mer was not protein-bound, versus 23% unbound for the 13mer; data not shown). All ASO were stable on incubation in plasma at 37° C with minimal detectable degradation for at least 48 h, but on incubation in hepatocyte extract for 24 h the MTO but not the MTPS was predominantly cleaved into monomers, as expected based on the nuclease-sensitivity of the phosphodiester linkage in the MTO (17,18) (data not shown).

**Figure 6.**
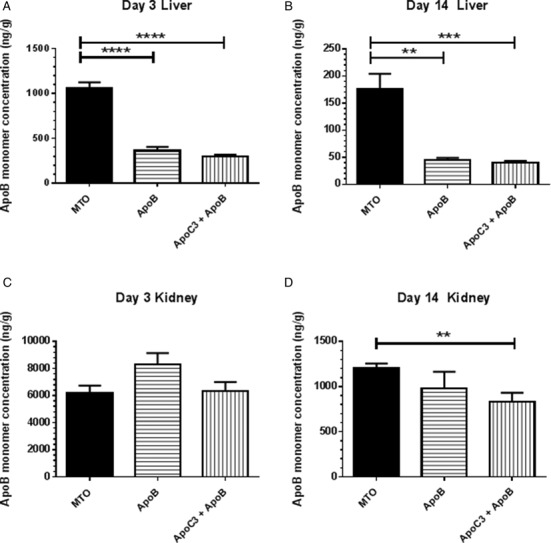
*In vivo* biodistribution of ApoB monomer after MTO or monomer dosing. The tissue concentrations of ApoB monomers in the mice shown in Figure 4A,B were measured using a novel hybridization assay at Day 3 (**A**, **C**) and Day 14 (**B**, **D**) in the liver (A, B) and kidney (C, D). The MTO delivered ≈4X more monomer into the liver compared to mice dosed with monomer, while delivering comparable concentrations of monomer into the kidneys. The second of two experiments performed with similar results is shown. **P < 0.005; ***P < 0.0005; ****P < 0.00005.

To determine the kinetics of cleavage of the MTO phosphodiester linker *in vivo*, and the biodistribution of the various ASO, we developed hybridization assays using specific oligonucleotide probes to measure the tissue concentrations of the ApoB/ApoC3 heterodimers (Table 2) and the ApoB monomer in liver and kidney tissue (Figure 6). In both the liver and kidney the concentration of the heterodimer MTPS was similar and declined slowly, but in contrast the heterodimer MTO was very rapidly cleaved, with a 93% reduction compared to the MTPS concentration in the liver already by the 3 day time point (Table 2). By day 14, the heterodimer concentration in the kidney and liver was reduced to about 25% in the tissues of the MTPS-treated mice, but was barely detectable for the MTO-treated mice, indicating nearly complete cleavage to monomer only for the MTO (Table 2).

**Table 2.** Tissue concentration of uncleaved heterodimer

Tissue	ASO	Day 3 conc. (ng/g; ± std. error)	Day 14 conc. (ng/g; ± std. error)
Kidney	MTPS	13 909 (± 3562)	3359 (± 969)
	MTO	7236 (± 1030)	24 (± 2)
Liver	MTPS	11 937 (± 1200)	2760 (± 764)
	MTO	846 (± 65)	35 (± 3)

The liver concentrations of the ApoB monomer in the MTO-treated mice are ≈4-fold higher compared to the monomer-treated mice at both day 3 and day 14, confirming our hypothesis that the MTO design provides a pharmacokinetic benefit over the administration of conventional ASO monomers and demonstrating that this benefit is sustained for at least 2 weeks (Figure 6). In sharp contrast to the increased delivery of monomer into the livers of MTO-treated mice, the MTO did not provide any significant increase in monomer delivery into the kidneys at the day 3 time point (Figure 6). The ApoB monomer concentrations in both the liver and kidney decreased by ≈80% between 3 and 14 days after treatment with MTO or monomers (Figure 6A–D).

## DISCUSSION

There has been much excitement in the promise of RNA therapeutics for improving human health. Along with this, there is great interest in approaches to improve ASO potency and enable dose reduction. We now demonstrate that MTO represents a simple ASO design compatible with shortmer and gapmer approaches, requiring no special chemistry or conjugation steps, which improves plasma protein binding with a concomitant increase of at least 4-fold in peripheral tissue uptake and *in vivo* potency. Based on these results, the same approach should also increase the efficacy of mixmer ASO. At present, all of the ASOs in active clinical development are designed to target single mRNAs. The MTO design enables the targeting of two different mRNAs with a single molecule, which should greatly expand the potential applications for ASO development by enabling the targeting of more than one gene in a pathway, or multiple disease-inducing genes in different pathways (Figure 1).

We hypothesize that at least two different factors may contribute to the improved *in vivo* efficacy of the MTO design compared to others. First, compared to the standard monomer design the increased plasma protein binding of the longer MTO should reduce renal clearance, thereby making more oligo available for uptake into the intended target tissues, as demonstrated in the liver, but also anticipated in other peripheral tissues. Since the affinity of ASO for plasma proteins is relatively low, bound ASO are still available for cell uptake via higher affinity cell surface proteins, by which means ASO are thought to be transported into a series of endosomal compartments (10,19). This hypothesis is supported by our finding that the MTO improved the concentration of monomer delivered into the liver by ≈4-fold (Figure 6), which is comparable to the 4- to 5-fold increase in *in vivo* potency, and also to the reduction in the percentage of free (unbound) ASO in plasma. Thus, MTO appears to function as a pro-drug for ASO, and the increased potency of MTO compared to monomer can be accounted for by the improved biodistribution into peripheral tissues, resulting in an increased efficacy and prolonged duration of action compared to standard short ASOs.

Second, we show that the increased *in vivo* efficacy of the MTO compared to the MTPS is due to the presence of the nuclease-sensitive linker in the MTO that enables its endosomal cleavage into monomers in contrast to the uncleavable MTPS. The fact that MTPS and MTO are equally effective upon transfection into cells demonstrates that there is no inherent defect in the ASO functional capacity of the MTPS when it is delivered into the cells by a route that bypasses the endosomes (i.e. transfection). It is notable that the potency advantage of the MTO over MTPS is only evident when they are assayed by free uptake (data not shown) or *in vivo*. In these experimental settings where MTO is more effective than MTPS, cell uptake is mediated via endosomal uptake and efficacy presumably requires endosomal escape, not withstanding the fact that the mechanisms of ASO uptake in these latter two systems may well differ (10). Endosomal escape is thought to be a rate-limiting step in the efficacy of ASO administered by either free uptake or *in vivo*. The great majority of intracellular ASO is thought to be trapped in nonfunctional endosomal/lysosomal compartments, with a very small fraction of ASO that either bypasses or exits the endosomes to enter the functional intracellular compartments where RNA function can be blocked, starting with the cytoplasm, from which free diffusion into the nucleus is thought to occur. The mechanism of ASO escape from endosomes is unclear, but has been hypothesized to involve transient discontinuities in the lipid bilayer during the vesicular budding and fusion events of endosome trafficking (19,20). Further studies will be required to determine whether longer ASO with more negative charges are less able to exit or bypass the endosomal compartments and enter the functional intracellular compartments compared to much shorter monomer ASO with a smaller net negative charge.

Several other approaches have been reported to improve ASO efficacy, some of which may be complementary to the MTO design. For example, a recent study reported markedly improved activity of ASO in the liver by conjugation of a triantennary *N*-acetyl galactosamine (GalNAc) high affinity ligand for the hepatocyte-specific asialoglycoprotein (20). It is likely that the efficacy of short ASO GalNAc conjugates for liver targets could be further improved by the adoption of the MTO design. Another PS ASO design reported to have improved activity compared to standard PS ASO is the ‘gene silencing oligonucleotide’ or GSO, in which two PS ASO are conjugated together by the 5′ ends (leaving two free 3′ ends) (21). Since the GSO design uses a non-cleavable linker, its reported efficacy *in vivo* presumably results from a different mechanism of action than the MTO.

The MTO design can be used to target any RNA, including for example, mRNA, miRNA and/or lncRNA. Compared to current short ASO designs, MTO should enable reduced dose levels and frequency of administration, which could have potential for broad experimental and human therapeutic applications. Nevertheless, it must be categorically stated that this paper is not reporting on animal toxicity. Therefore, claims of efficacy must be judged against the absence of these data; it is not possible to fully evaluate the clinical potential of these novel molecules until animal toxicity has been evaluated, which we hope to do at a later date.
